# Testing Protein Stress Signals in Peripheral Immunocytes Under the Same Treatment Capable of Decreasing the Incidence of Alzheimer’s Disease in Bladder Cancer Patients

**DOI:** 10.3390/cimb47060392

**Published:** 2025-05-26

**Authors:** Benjamin Y. Klein, Ofer N. Gofrit, Charles L. Greenblatt

**Affiliations:** 1 Department of Microbiology and Molecular Genetics, Hebrew University Medical School, Jerusalem 91120, Israel; charlesg@ekmd.huji.ac.il; 2 Department of Urology, Hadassah University Medical School, Ein-Karem, Jerusalem 91120, Israel; ogofrit@gmail.com

**Keywords:** BCG vaccine, peripheral blood mononuclear cells, unfolded protein response, endoplasmic reticulum stress, cell signaling, immunoelectrophoretic

## Abstract

Several studies showed that the incidence of Alzheimer’s disease (AD) is significantly lower in patients with non-muscle invasive bladder cancer (NMIBC) treated with intravesical bacillus Calmette–Guérin (BCG) instillations compared to treatment by alternative methods. Hypothetically, failure to clear misfolded and aggregated proteins (i.e., beta-amyloid) in AD brains and peripheral blood mononuclear cells (PBMCs) implicates BCG in upgrading the unfolded protein response (UPR). To test this hypothesis, pre- versus post-BCG PBMC proteins of the UPR pathway were compared in six NMIBC patients by capillary immunoelectrophoresis on an Abby instrument. PERK, the endoplasmic reticulum (ER) resident kinase, a stress-activated sensor, and its substrate alpha component of the eIF2 translation factor (eIF2a) complex inactivation were considered as potentially proapoptotic via a downstream proapoptotic transcription factor only if persistently high. GAPDH, a glycolytic marker of innate immunocyte training by BCG, and eight other UPR proteins were considered antiapoptotic. Summation of antiapoptotic %change scores per patient showed that the older the age, the lower the antiapoptotic %change. Higher antiapoptotic scores were observed upon a longer time from BCG treatment (with the exception of the patient in her ninth decade of life). Studies with more individuals could substantiate that BCG enhances the antiapoptotic aggregate-clearance effect of the UPR in PBMCs of NMIBC patients, which hypothetically protects brain cells against AD.

## 1. Introduction

### 1.1. Alzheimer’s Disease Prevention by Bacillus Calmette–Guérin

High-grade superficial or recurrent low-grade bladder cancer is treated by transurethral tumor resection and adjuvant intravesical instillations of the bacillus Calmette–Guérin (BCG) vaccine. This treatment effectively reduces tumor relapse compared with tumor resection only [1]. The sequence of the immunological events that follow BCG instillations has not been completely deciphered yet, but a general scheme of participating immunocytes has been described [2], where the primary responding cells are granulocytes, monocytes, macrophages, and natural killer cells. Subsets of T cells are subsequently recruited by various cytokines secreted mainly by the initial polymorphonuclear neutrophil responders, and the T cells are stimulated by antigen-presenting cells to recognize tumor-specific antigens [3]. Neutrophils have also been shown to accumulate in the bladder urine following BCG instillation and are responsible for the effective anti-tumor activity of BCG [4]. This connects to the ability of BCG to induce innate immune cell “training”, a phenomenon that epigenetically generates a non-specific memory, upgrading the cytokine response to subsequent encounters with BCG (and other pathogens) of these innate immunocytes [5,6]. BCG is an attenuated live bovine tuberculosis mycobacterium used globally since 1921 as an anti-tuberculosis vaccine [7]. As newborns have not been BCG-vaccinated prophylactically against tuberculosis in the USA, as opposed to Sweden, we were impressed by the low incidence of Alzheimer’s disease (AD) in Sweden compared to the USA and hypothesized that BCG vaccination may be one factor responsible for this difference [8]. The long-lasting practice for decades of treatment of non-muscle invasive bladder cancer (NMIBC) by BCG instillations was employed to analyze the incidence of AD during the recent decades in BCG-treated versus other treatments of NMIBC patients. The results indicated that intravesical BCG treatment reduced the incidence of AD by up to fourfold relative to control patients [9]. Since then, variable levels of effectiveness of BCG against AD in NMIBC patients have been demonstrated [10]. However, vaccinations against other pathogens have also been shown to reduce the incidence of AD [11,12]. This indicates that the prevention of AD by BCG does not depend on immunity against specific antigens as in the case of NMIBC but is due to biochemical changes that immunocytes undergo through immunization stimulus, for example, the epigenetic “training” of innate immunocytes [13,14]. A significant feature of AD is the failure to control the misfolding and aggregation of newly synthesized proteins such as beta-amyloid and its derivatives [15,16,17]. The successful clearance of beta-amyloid plaques by designated antibodies is not always beneficial to the patient’s clinical status [18,19,20], indicating that factors upstream to the normal biochemical clearance are responsible for plaque accumulation. This implies that the unfolded protein response (UPR) in the brain, the immediate upstream responding pathway to endoplasmic reticulum (ER) stress, is chronically less efficient than normal [21].

### 1.2. Involvement of the Unfolded Protein Response in Alzheimer’s Disease

**Figure 1:**
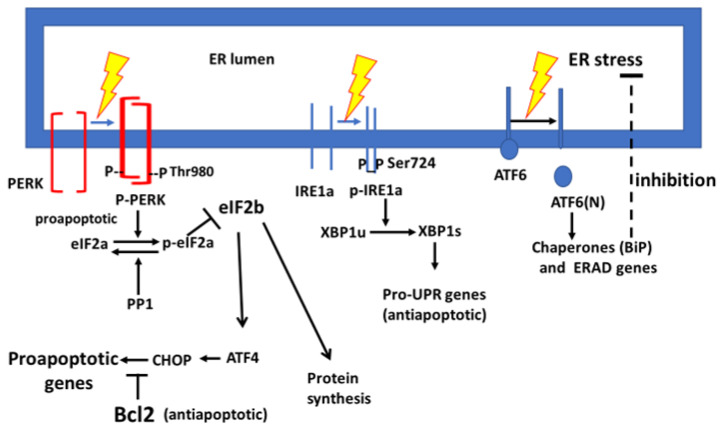
The response of ER stress of three ER membrane resident stress sensors and downstream responses. The designation of p-PERK, p-eIF2a, and CHOP as proapoptotic relates to prolonged ER stress.

We raised the question of whether BCG treatment can significantly influence the signaling proteins of the UPR in peripheral blood mononuclear cells (PBMCs), influencing brain tissue and postponing or protecting against AD. The UPR pathway consists of ER stress-sensing proteins that transduce cap-dependent translation shutdown, i.e., for most cellular mRNA. A minority of cap-independent mRNA translation uses internal ribosome entry sites (IRESs). During translation shutdown, activation of transcription factors that activate the expression of chaperones and aggregate clearance factors correct misfolded proteins and degrade the aggregates [22,23,24]. The UPR signaling pathway has been reviewed in general [25] and is related to AD pathology [26,27] and can be briefly summarized. Newly synthesized proteins translocate into the ER for quality control of their proper and functional folding by chaperones such as the immunoglobulin binding protein (BiP). Upon ER overflow with new translation products, the free excess proteins unmatched by chaperones cause ER stress, sensed by stress sensors on the ER membrane. One of these ER stress sensors, the protein kinase R (PKR)-like ER-resident kinase (PERK), causes PERK monomers to dimerize and undergo phosphorylation that opens a kinase domain at the cytosolic face of the ER membrane [24,28]. This kinase domain specifically phosphorylates the eIF2a component of the eIF2 translation factor complex on its Ser51, and this inhibits all cap-dependent mRNA translation until ER stress is resolved [25]. ER stress also activates the transcription factor 6 (ATF6). This stress sensor migrates laterally in the membrane towards the Golgi apparatus [25]. It then undergoes an intramembranous cleavage [29] in which the cytosol-facing N-terminal fragment becomes a transcription factor (ATF6[N]). Its nuclear transcription program includes activation of the BiP and the X-Box protein 1 (XBP1) genes and ER stress-associated degradation (ERAD) factors that remove misfolded proteins from the ER for degradation at the proteasome [30]. Old astrocyte-specifically induced substance (OASIS) is cleaved similarly to ATF6 and is synergistic with it [31]. Inositol-requiring enzyme 1 (IRE1a) dimerizes or oligomerizes and becomes phosphorylated in response to ER stress to uncover an RNA endonuclease at its cytosolic face that excises 26 nucleotides of the XBP1 mRNA [25]. This unconventional mRNA splicing translates into the spliced XBP1 variant (XBPs), which acts as a transcription factor activating the expression of the BiP gene [29,30,32]. These are the major components of the UPR cycle of protein overload that activate the ER stress sensors. The stress-induced expression of ER chaperones controls the folding of proteins and removes misfolded proteins from the ER until the next ER stress cycle. In case of failure to clear misfolded proteins from the ER, the activated PERK (p-PERK) and its phosphorylated substrate (p-eIF2a) increase chronically to stimulate the activated transcription factor 4 (ATF4) expression [33]. The C/EBP homolog protein (CHOP) activation by ATF4 inhibits the expression of cell survival proteins; therefore, CHOP promotes the expression of cell death proteins (Figure 1). Thus, chronic UPR failure may cause apoptosis of brain glia and astroglia on which neuron energy metabolism depends [34,35,36]. An interesting question is how peripheral immunocytes react to the two divergent targets, AD versus NMIBC pre- and post-BCG therapy. Here, we focus mainly on AD. The brain is infiltrated by immunocytes, which may contribute to dementia during aging [37,38,39]. We hypothesize that PBMCs with enhanced UPR induced by BCG can replace pre-BCG brain-infiltrating immunocytes and, in this way, protect against AD. The accumulation of neutrophils in the urine of NMIBC patients treated with BCG, as described above, may reflect granulopoiesis whose results should be detectable in the PBMCs. The task of proving this hypothesis is formidable; we thus undertook a simple pilot study to reveal the impact of BCG intravesical instillations on the UPR in PBMCs.

## 2. Materials and Methods

### 2.1. Treatment Protocol

Patients with high-grade NMIBC were considered candidates for BCG therapy by six weekly intravesical instillations of OncoTICE BCG (Merck, Kirkland, QC, Canada) in 50 cubic centimeters (cc) of normal saline (0.9% NaCl), following peripheral blood sampling for PBMC isolation. For age, gender, and time of blood sampling post-BCG therapy, see Table 1. This study was approved by the committee on research involving human subjects of the Hadassah Hebrew University Hospital (application 0798-21 HMO, 3rd year extension up to 27 February 2025). All patients provided informed consent.

### 2.2. Peripheral Blood Mononuclear Cell Sampling

Before and after intravesical BCG instillation, venous blood was drawn and separated on Ficoll Histopaque-10771 (Sigma, Rehovot, Israel); centrifuged at 1200× *g* for 30 min. PBMCs at the interphase with dilute upper-phase plasma were aspirated, washed in RPMI-1640 (R8758, Sigma), suspended in cold DMSO (D2438, Sigma), and 20% cold fetal calf serum (FCS) (F9665, Sigma) and gradually frozen to −170 °C, to be kept in liquid nitrogen until further use.

### 2.3. Thawing of Peripheral Blood Mononuclear Cells and Protein Extraction

PBMCs in frozen vials were rapidly thawed in a water bath at room temperature until the frozen suspension reached 0 °C. Cells were diluted in 20 volumes of RPMI, spun for 10 min, 300× *g* at 4 °C, and washed twice in cold plain Dulbecco’s phosphate-buffered saline (D8537, Sigma) to remove all cryopreservation solution and FCS traces. The cell pellets were suspended in a 0.2 mL ice-cold Bicine/CHAPS detergent lysis buffer kit containing inhibitors of proteases and phosphatases (CBS403, ProteinSimple, San Jose, CA, USA) designed to minimize nuclear lysis. The cell suspension was incubated on ice for 30 min and spun for 30 min at 10,000× *g* at 4 °C. Protein supernatants were frozen at −20 °C until further use.

### 2.4. Preparation of Immune-Electrophoresis Samples

Samples were prepared using a separation module kit for a molecular weight range of 12–230 kDa MW for a 25-capillary cartridge (SM-W004) compatible with an Abby instrument (see ProteinSimple website for the Abby instrument). A master-mix solution was prepared according to the producer’s instructions using a standard pack from the kit that provides dithiothreitol, a proprietary master mix compound, and a 5× concentrated sample buffer. The ready-to-use master mix was prepared such that for one capillary, a 0.6 μL master mix was added to 2.4 μL of protein solution (total volume/sample = 3 μL, with 0.8 μg protein), boiled at 95 °C for 5 min and loaded on a producer-designed plate in a row of 24 wells for the samples, and 1 well for the supplied biotinylated molecular weight ladder. The kit’s detection module (mostly anti-rabbit DM-001) contained the secondary antibody horse radish peroxidase (HRP) conjugate, the antibody diluent, which also serves as a blocking buffer, luminol, and hydrogen peroxide. The rest of the separation module of the kit contains a wash buffer, plates, and capillary cartridges. We also used a total-protein detection module (DM-TP01) in a Re-Plex format, in which, after the regular immune electrophoresis, the antibodies underwent in-capillary stripping and a catalytic reagent biotinylated the total in-capillary proteins detected by streptavidin. The primary antibodies were purchased separately from other suppliers. From the plate loading onto the instrument to the end of the procedure, all the described electrophoretic and catalytic steps were automatically performed and computer-recorded in run files, without human intervention. The run protocol included the following steps: a separation time of 25 min, 375 V, primary and secondary antibodies 30 min each and detection time of 30 min.

### 2.5. Antibodies

Anti-PERK rabbit monoclonal antibodies (R-mAb) from Cell Signaling Technology (CST) (Danvers, MA, USA), catalog number (#) 3192, included anti-phospho-PERK pThr980 R-mAb CST #3179, anti-IRE1a R-mAb CST #3294, anti-phospho-IRE1a p-S724 R-polyclonal Ab Novus Biologicals (NB) (Centennial, CO, USA) #NB100-2323, anti-BiP R-mAb CST #3177, anti-glyceraldehyde phosphate dehydrogenase (GAPDH) R-mAb XP CST #5174, anti eIF2a mouse-mAb CST #2103, anti-phospho-eIF2a Ser51 R-mAb CST #9721, anti ATF6 rabbit polyclonal Ab NB #NBP1-75478, anti-XBP1 R-polyclonal Ab NB #NBP1-77681, in addition to anti-OASIS R-polyclonal Ab NBP1-31017 NB, anti-GADD153/CHOP R-polyclonal Ab NBP2-13172 NB, anti-Bcl-2 mouse mAb #15071 CST, and anti-Bcl-2A1 mouse mAb NB100-58070 NB. Antibodies were diluted 1:100 in the diluent solution provided in the detection module of the electrophoresis kit (ProteinSimple).

### 2.6. Analysis of the Results

The Abby instrument provided results by presenting protein curve peaks (detected by antibodies), and the quantities of proteins under the curves were recorded in the run files. The arbitrary numbers of the relevant antigen sizes were copied from the original run files of the instrument and presented in the Supplementary Section (Table S1). The relevant protein peaks were expressed as arbitrary unit numbers per total protein values obtained by a designated kit for in-capillary protein quantitation (DM-TP01, ProteinSimple). The values of the total protein along each capillary were recorded by the instrument and were copied into an Excel file, to which the antigen density of peaks from respective capillaries is related.

### 2.7. Presentation of the Results

The arbitrary antigen densities are mostly presented as a fraction of the total protein density, obtained in arbitrary numbers of chemiluminescence, as detected by the Abby instrument. These relative antigen (signaling protein) densities are presented per patient, as pre- versus post-BCG treatment values. The illustration is either by comparative bar graphs or linear or other regression in which the regression lines contain pre-BCG results in blue versus post-BCG results in red. The correlation coefficients of each regression are provided using PowerPoint software, and their statistical significance is computed via the statistical package for the social sciences. The regression lines express the relation between enzymes on the *x*-axis and expected responding protein substrates on the *y*-axis. On top of some regression lines, the effect of the BCG-reinforced vaccine course is overlaid by an arrow pointing up or downwards for the substrate (*y*-axis) or the responding protein, and horizontally for the enzyme or activation protein (*x*-axis). Each pair of arrows represents a respective patient number. A summary table of positive or negative effects of BCG is presented separately for all detected antigens. A positive (+) or negative (−) score is added as a quantitative evaluation that indicates the potential survival or apoptotic direction of each patient’s total UPR. The scores (total %changes) of each patient are recorded in the bottom rows.

### 2.8. Calculation of the Score

For each UPR protein factor per patient, the pre-BCG value is subtracted from the post-BCG value; thus, if BCG increases or decreases the protein/total protein ratio, the result is positive (gain) or negative (loss) of expression, respectively. The expression gain is presented as the percentage gain incurred in the post-BCG results, and the loss is expressed as the percentage of the loss incurred in the pre-BCG result. Phosphorylation of PERK and eIF2a leads to CHOP expression, which stimulates cell death-inducing protein genes. Therefore, p-PERK, p-eIF2a, and CHOP are proapoptotic because if their phosphorylation persists, the cell will undergo apoptosis. The total proapoptotic effect by the tested UPR proteins is the sum of these three results. If one of these showed a negative proapoptotic value, it is considered a reduction in the proapoptotic effect and subtracted from the positive proapoptotic total. The opposite practice is considered for the rest of the nine antiapoptotic effects of the UPR and cell survival proteins. A third scoring value is a balance between pro- and antiapoptotic scores. The calculations are presented in the Supplementary Material Section (Table S2).

## 3. Results

### 3.1. Presentation of the BCG Impact on the UPR

BCG-induced changes for each UPR signaling protein are illustrated separately for patients in a horizontal row (Table 2). A scheme of the UPR landscape is presented in Figure 1.

To deal with patient heterogeneity, BCG-induced changes for each UPR protein are expressed by a numbered value in Table 2.

### 3.2. PERK Response to Endoplasmic Reticulum Stress After Bacillus Calmette–Guérin Treatment

**Figure 2:**
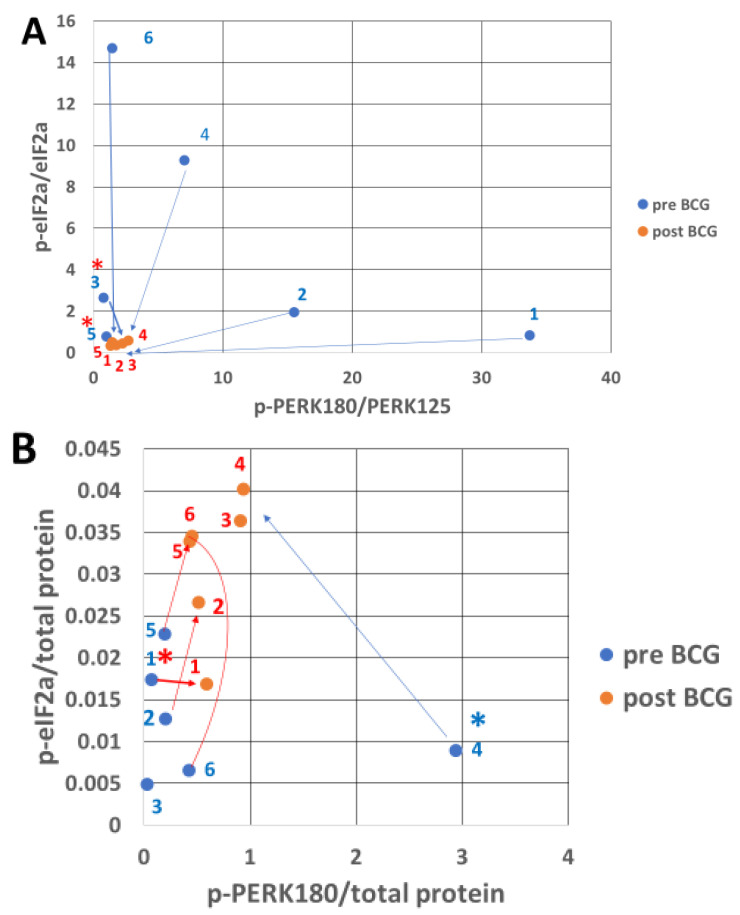
Inactivated eIF2a protein (p-eIF2a) abundance versus its activated kinase p-PERK abundance. Pre-BCG p-PERK (*x*-axis) is plotted against its responsive substrate pre-BCG p-eIF2a (*y*-axis, blue dots), each relative to its total pre-BCG abundance (**A**). The same relation is depicted for each patient post-BCG treatment in red. (**A**) Note the drastic reduction in the post-BCG ratios and the restriction to the lower left corner. For patients 3 and 5, the red asterisk denotes a slight increase in p-PERK expression; the mean p-PERK activity pre-BCG is 9.9139 and post-BCG is 0.218 (*p* = 0.094, n = 6) according to the *T*-test. The parallel pre-BCG expression of the inactive p-eIF2a form is 5.042 and becomes 0.4437 post-BCG (*p* = 0.038, n = 6) by *T*-test. (**B**) p-PERK and p-eIF2a are related to the total cell protein for each patient pre- versus post-BCG treatment. The mean p-PERK/total protein ratio is 0.4107 pre and 0.6383 post-BCG (*p* = 0.459, n = 6) by *T*-test. In parallel, the pre-BCG mean p-eIF2a/total protein level is 0.0075 (high activity) versus 0.0213 post-BCG, demonstrating lower activity relative to the reduced protein translation (*p* = 0.019, n = 6) according to the *T*-test. Exceptional results shown by the blue and red asterisks indicate heterogeneity in patient response. See Table S1 for data.

Figure 2A shows a high ratio of activated PERK (p-PERK, Thr980) relative to the total PERK before BCG treatment in patients 1, 2, and 4. BCG treatment has drastically decreased the p-PERK/PERK ratio due to increased denominator (total PERK, Table S1). When total PERK is replaced by total protein as the denominator (p-PERK/total protein, Figure 2B), BCG treatment increases the total protein-normalized p-PERK in patients 1, 2, 3, 5, and 6 compared to the respective untreated PBMC samples. The total eIF2a, an alpha component of the eIF2 translation factor complex, shows an inactivating phosphorylation of p-eIF2a relative to total eIF2a in untreated patients 2, 3, 4, and 6 (Figure 2A). When p-eIF2a is related to total protein, instead of total eIF2a protein levels, there is a higher abundance of inactivated eIF2a (p-eIF2a/total protein) in samples of BCG-treated patients 2, 3, 4, 5, and 6 (Figure 2B). These results indicate that BCG increases the total protein, but it increases p-PERK and p-eIF2a abundance more than the respective protein relative to most untreated patient samples (Table S1). Mean PERK is activated by BCG (to p-PERK), although insignificantly. Nevertheless, BCG significantly inactivates the PERK substrate p-eIF2a. 

### 3.3. CHOP and BCL2 Expression After Bacillus Calmette–Guérin Treatment

**Figure 3:**
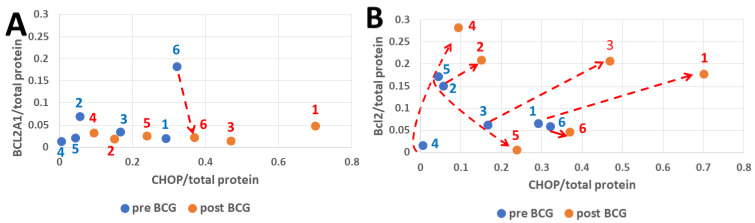
The impact of BCG on Bcl2A1 and Bcl2 cell survival proteins in parallel to the proapoptotic transcription factor CHOP. In all the patients, the CHOP/total protein abundance ratio increased by treatment with BCG (*y*-axis red versus blue dots (and red dashed arrows) panels **A** and **B**). The range of Bcl2A1 changes (*x*-axis, **A**) is small, except for patient 6. The range of Bcl2 changes is much more pronounced (**B**) than seen for Bcl2A1 (**A**). The mean activity of CHOP relative to total protein is 0.14796 pre- and 0.33726 post-BCG, which is significant (*p* = 0.0022, n = 6) according to a paired *T*-test. The mean Bcl2 activity is 0.08712 pre- and 0.15426 post-BCG, insignificant according to a *T*-test (*p* = 0.315, n = 6) as patients 5 and 6 have lost activity. By *T*-test, Bcl2A1 showed insignificant mean activity after BCG therapy (*p* = 0.342, n = 6).

The CHOP, Bcl2A1, and Bcl2 response to BCG treatment is shown in Figure 3. The A1 variant of the cell survival protein Bcl2 shows a limited response to BCG (Figure 3A). Canonical Bcl2 responded positively to BCG at a variable range in patients 1, 2, 3, and 4, and decreased in patients 5 and 6. The response of CHOP to BCG showed a variable range, increasing in all patients as seen in Figure 3A,B. Thus, in patients 1, 2, 3, and 4, Bcl2 poses a counteracting challenge to the potential cell death inducer CHOP. CHOP expression is an event downstream of the activation of PERK (p-PERK) and inactivation of its substrate eIF2a (p-eIF2a).

### 3.4. The Response of IRE1a and Its XBP1 Substrate to Bacillus Calmette–Guérin

**Figure 4:**
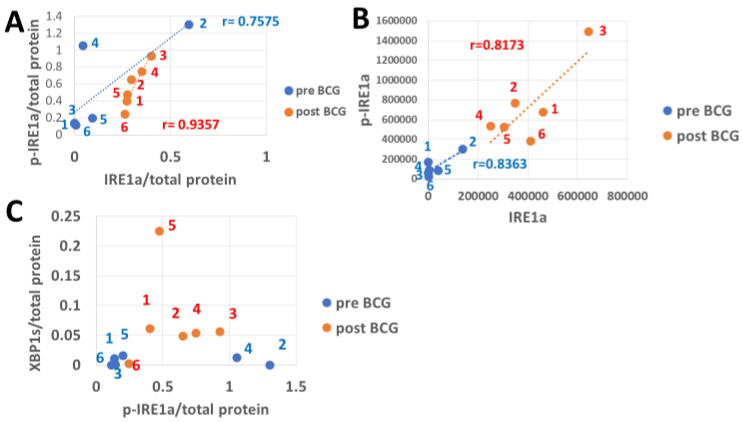
BCG activation of stress response via the IRE1a stress sensor. The abundance of IRE1a for six BCG-treated patients on the *x*-axis matched the resulting p-IRE1a depicted on the *y*-axis. P-IRE1a in patients 2 and 4 is downregulated (**A**). (**B**) Pre- and post-BCG dot scatter plots represent absolute values of p-IRE1a abundance (*y*-axis) matched with those of IRE1a abundance (*x*-axis). (**C**) The spliced XBP1 protein (XBP1s/total protein abundance ratio) on the *y*-axis is matched with p-IRE1a/total protein on the *x*-axis. The mean IRE1a/total protein pre-BCG increased significantly from 0.124 to 0.3099 post-BCG (*p* = 0.043, n = 6) according to the *T*-test, both on the *x*-axis (**A**). The mean p-IRE1a/total protein abundance ratio pre-BCG increased insignificantly from 0.492 pre-BCG to 0.575 post-BCG (*p* = 0.35, n = 6), both on the *y*-axis, (**A**). Responding to p-IRE1a, the XBP1/total protein mean value grew significantly from 0.00657 pre-BCG to 0.07435 post-BCG (*p* = 0.03445, n = 6, panel **C**) according to the *T*-test. Dashed lines indicate by color the regression lines.

Phosphorylation of the ER stress sensor IRE1a is activated by ER stress to yield p-IRE1a (Figure 4A,B), which is followed by its downstream splicing effect on unspliced XBP1 (XBP1u) mRNA to obtain the spliced translation product XBP1s (Figure 4C). The low abundance of IRE1a corrected by total protein is congruent with the low abundance of p-IRE1a in patients 1, 3, 5, and 6 before BCG treatment, and so is the high abundance of IRE1a in patient 2. For patient 4 pre-BCG treatment, their low abundance resulted in a high activated p-IRE1a/total protein abundance ratio, lowering the correlation coefficient versus the BCG-treated patients, where r = 0.7575, *p* = 0.0405, versus r = 0.9357 and *p* = 0.003, respectively. Of note, the pre-BCG total p-PERK abundance was higher than that of post-BCG in patient 4 when normalized to total protein (Figure 2B), inversely to the IRE1a in which a relatively low total protein denominator leads to high p-IRE1a value on the *y*-axis (Figure 2A, see Discussion for a possible mechanism). The levels of IRE1a conversion to p-IRE1a at absolute abundance (Figure 4B) were roughly congruent in all patients for both the pre- and post-BCG treated groups (r = 0.8363, *p* = 0.019 and r = 0.8173, *p* = 0.023). The ratios of IRE1a to p-IRE1a conversion were similar, but the quantities were higher after BCG treatment for patients 1, 3, 4, 5, and 6. For patient 2, they were equal (Figure 4B). The mean pre-BCG was 0.1749 pre- and 0.6136 post-BCG (*p* = 0.016, n = 6). The abundance of XBP1s normalized to total protein has increased in patients 1, 2, 3, and 4 after BCG treatment, which was congruent with the levels of p-IRE1a only for patients 1, 3, and 5, while the increase in patients 2 and 4 was much less than expected from the high levels of the pre-BCG p-IRE1a (Figure 4C).

### 3.5. The Impact of Bacillus Calmette–Guérin on BiP Expression in Response to Endoplasmic Reticulum Stress-Induced Transcription Factors

**Figure 5:**
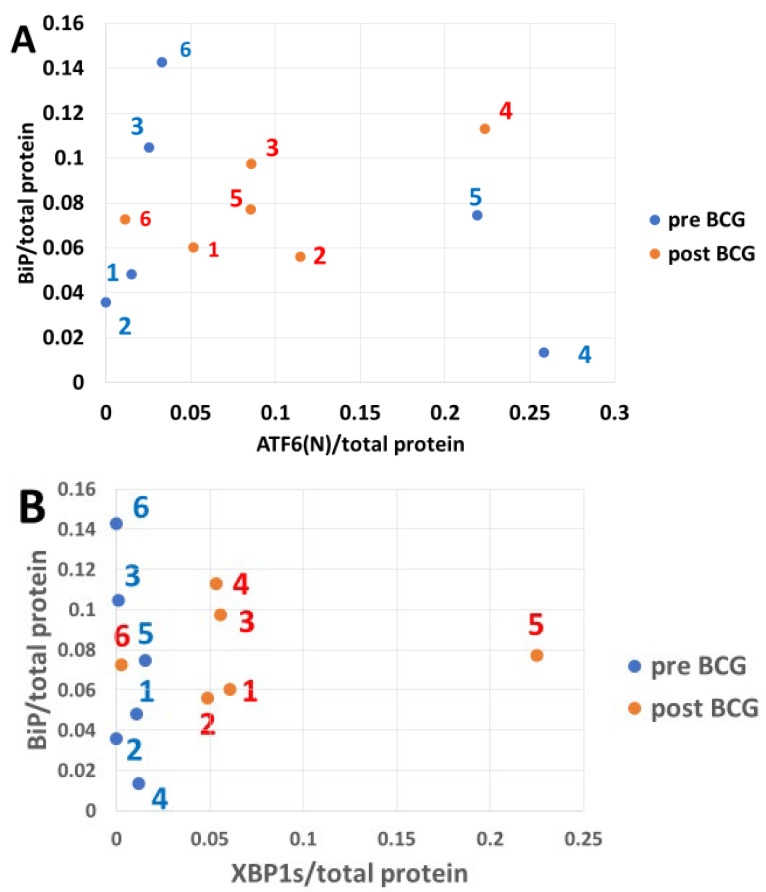

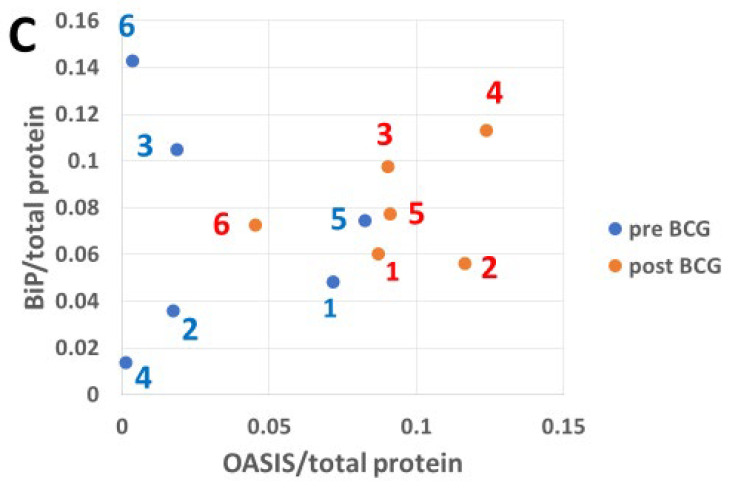
The effect of BCG on UPR transcription factor activation responding to ER stress sensors. BiP (*y*-axis) transcription target abundance plotted in comparison to that of ATF6(N) (**A**), XBP1s (**B**), and OASIS (**C**) (*x*-axis). Note the very low levels of pre-BCG XBP1s compared to ATF6(N). The mean ATF6(N)/total protein levels pre-BCG grew only slightly and insignificantly from 0.0917 to 0.0953 post-BCG (*p* = 0.46, n = 6, panel **A**) according to the *T*-test; only patients 1, 2, and 3 contributed to its growth. The mean BiP/total protein abundance ratio grew minimally from 0.0699 pre-BCG to 0.0794 post-BCG (by *T*-test, *p* = 0.343, n = 6, *y*-axis in panels **A**–**C**); only patients 1, 2, and 5 contributed to its growth. The mean OASIS/total protein grew significantly from 0.0353 pre-BCG to 0.0923 post-BCG (by *T*-test, *p* = 0.012, n = 6, *x*-axis panel **C**).

Figure 5 shows the pre- versus post-BCG levels of ATF6(N), XBP1s, and OASIS matched with levels of the BiP protein expression in response to these transcription factors. The pre-BCG abundance of ATF6(N) is substantially higher than that of XBP1s in patients 2, 3, 4, 5, and 6 by >10, >26, >21, >14, >2000 fold, respectively, and in patient 1, it is only >1.3 fold higher than the XBP1s. The pre-BCG abundance of OASIS was higher than that of XBP1s in patients 1, 2, 3, 5, and 6 by >6, >4800, >19, >5, and >2300 fold, respectively. Patient 4 was an exception in which OASIS abundance was 0.1 of that of XBP1s. These results are consistent with the delayed XBP1 protein expression compared with the faster expression of ATF6(N) [30], which is synergistic with OASIS. In all patients post-BCG treatment, XBP1 abundance was higher than that of the pre-BCG samples. The ATF6(N) abundance was reduced in patients 4, 5, and 6 and increased in 1, 2, and 3 after BCG treatment, indicating a BCG mitigating effect on ATF6(N). The abundance of OASIS increased after BCG treatment in all patients.

**Figure 6:**
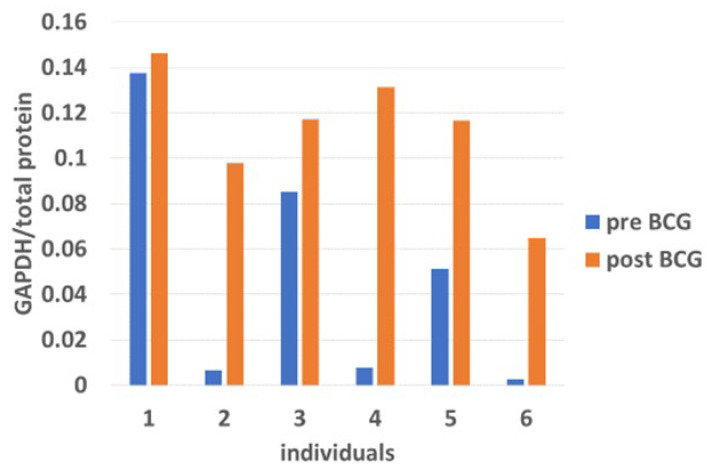
GAPDH levels increase in PBMCs in response to intravesical BCG instillations. GAPDH expression was elevated by BCG treatment in all patients. The mean GAPDH/total protein abundance ratio grew significantly from 0.04854 pre-BCG to 0.11238 post-BCG (*p* = 0.0061, n = 6, by *T*-test).

Expression of GAPDH, a representative enzyme of the glycolytic pathway, was variably upregulated in PBMCs of all patients by BCG therapy (Figure 6). This is consistent with converting anaerobic glycolysis to aerobic glycolysis by BCG, part of the innate immune training [40,41], and the metabolic change in activated adaptive immune cells [42]. GAPDH increased in PBMCs in response to intravesical BCG instillations. GAPDH, important for the glycolytic pathway, was elevated by BCG treatment in all patients. The variability of its increase reflects the heterogeneity between patients in converting from anaerobic to aerobic glycolysis in PBMCs.

### 3.6. Combining the Bacillus Calmette–Guérin Apoptotic and Antiapoptotic Effects

**Figure 7:**
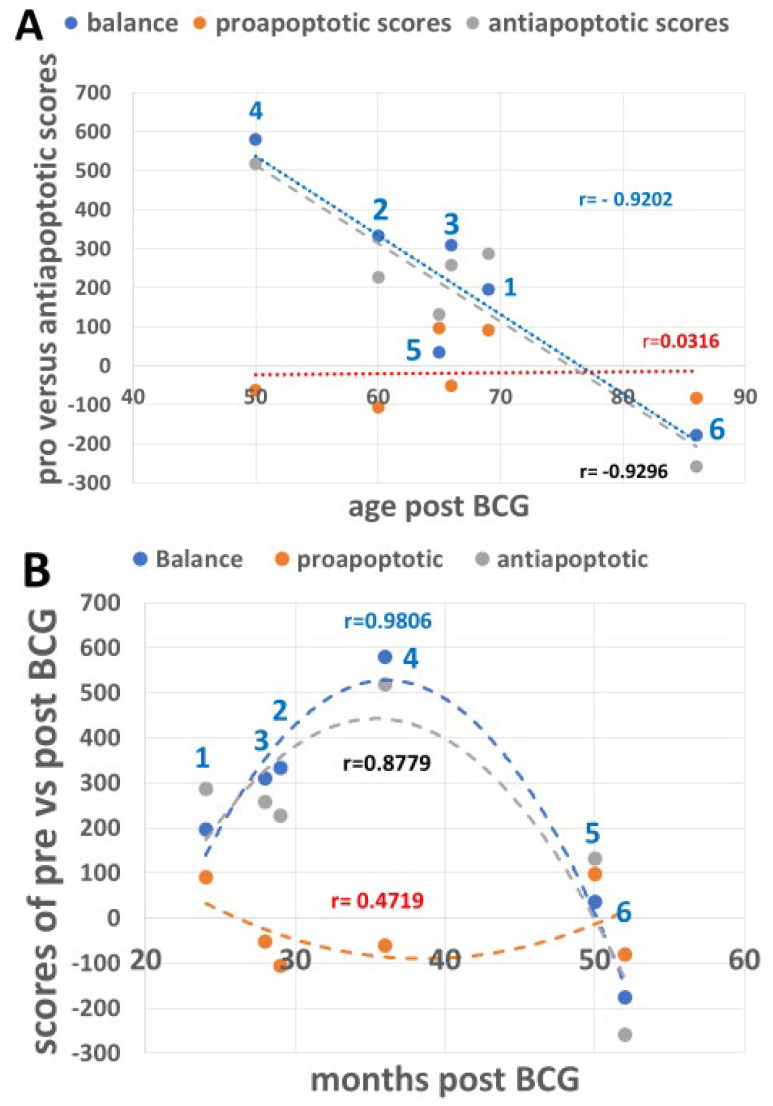
UPR signaling scores of BCG-induced percent changes versus patient age and months post-BCG treatment (Table 1). (**A**) The effect of age on the scores post-BCG treatment (from Table 2) resulting from the sum of percent changes of pro- versus antiapoptotic UPR signals and the balance between them (antiapoptotic r = −0.9296, proapoptotic r = 0.03162, for the balance between them r = −0.9202). The proapoptotic scores result from p-PERK and p-eIF2a related to total PERK and eIF2a, respectively. The difference between pro- and antiapoptotic correlations and between the balance and the proapoptotic correlation, according to Fisher’s r-to-z transformation test, is significant (*p* = 0.0388 and *p* = 0.0400, respectively). (**B**) The effect of time elapsed from BCG treatment (Table 1) to signaling score differences in PBMCs (Table 2) shows differences between the proapoptotic versus antiapoptotic values, with the best fit being a binomial correlation (proapoptotic r = −0.4719 versus antiapoptotic r = 0.8779, correlation difference *p* = 0.0213). The difference in apoptotic regression (r = −0.4719) from the regression of balance (r = 0.9806) is significant (*p* = 0.0005) in Fisher’s transformation. Dashed red, gray, and blue lines denote proapoptotic, antiapoptotic, and the balance between them, respectively.

**Figure 8:**
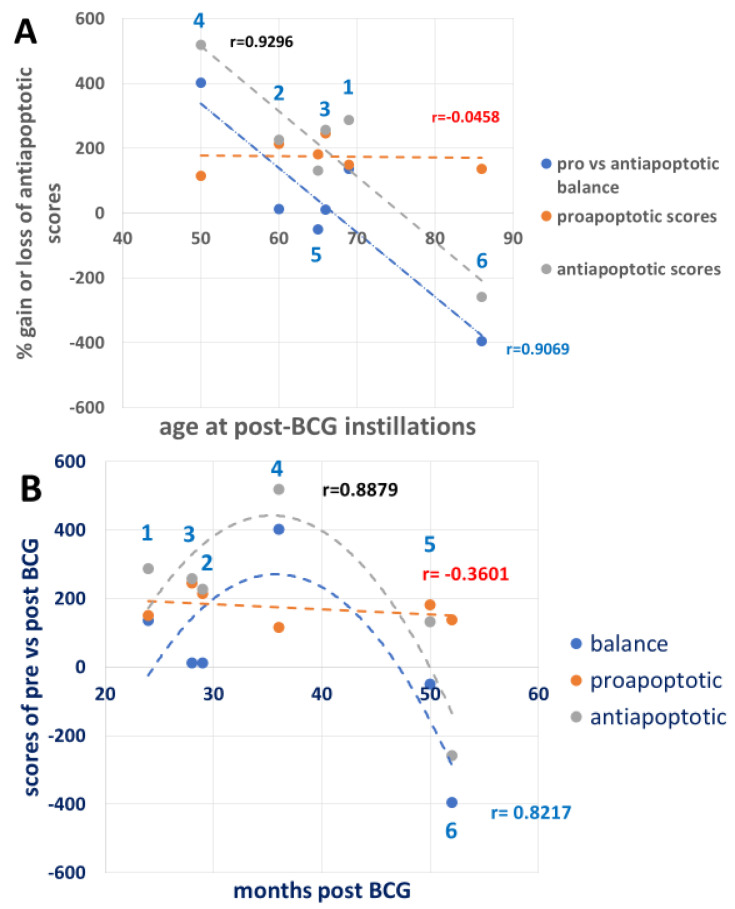
UPR signaling scores of BCG-induced percent changes versus patient age and months post-BCG treatment (Table 1). (**A**) Age post-BCG treatment effect on the scores taken from Table 3, in which the UPR proapoptotic signals are expressed as abundance relative to total cell protein. Note the elevation of the linear proapoptotic scores is still almost parallel to the *x*-axis, indicating that the proapoptotic scores are not affected by age or time post-BCG treatments. (**B**) The effect of the time interval between BCG therapy and the post-BCG treatment antiapoptotic scores. Note that the longer the interval, the higher the antiapoptotic scores of patients 1, 2, 3, and 4. The proapoptotic scores are less affected by the elapsed time. The antiapoptotic best fit regression is binomial, and the proapoptotic regression is linear. Dashed red, gray, and blue lines denote proapoptotic, antiapoptotic, and the balance between them, respectively.

The BCG antiapoptotic effect is a combination of %gain/loss of p-eIF2a, p-PERK, and CHOP depicted in Table 2, in which p-eIF2a is related to total eIF2a and p-PERK to total PERK as shown in Figure 2A and not when related to total protein (Figure 2B). Figure 7A shows that the antiapoptotic effect of BCG (combining the rest of UPR proteins in Table 2, line 14, related to the time elapsed from the start of BCG treatment shown in Table 1) decreases with age and stays above the *y*-axis 0-line until the 8th decade and falls below it for one patient in the 9th decade. In contrast, the proapoptotic line stays stable as a flat line close to the 0 line. Figure 7B shows the effect of BCG on the anti- and proapoptotic scores as a measure of time elapsed from the post-BCG harvest of PBMCs, along the *x*-axis of the short time scale (month intervals in Figure 7B versus age in years in Figure 7A). The time-dependent impact of BCG on the antiapoptotic effect is reminiscent of the time required for BCG to convert anaerobic to aerobic glycolysis in type 1 diabetics [41]. Yet, for the oldest patient (86 years old), 52 months of BCG treatment were insufficient to raise the BCG-induced antiapoptotic effect above the *y*-axis 0 line. These results may indicate that the younger the patient, the higher the BCG-induced antiapoptotic score, as its level requires about 3 years to show the full BCG impact. Unlike in Table 2, the p-PERK and p-eIF2a abundances are expressed per total protein, in Table 3. This brings the proapoptotic curve (Figure 8A) to a higher level about the *y*-axis than in Figure 7A while maintaining its flatness as in Figure 7A. These results indicate that in the present experiment, the proapoptotic potential with BCG treatment does not change with advancing age as the antiapoptotic score does.

## 4. Discussion

The rationale for examining the impact of BCG on the signaling cascade of UPR, which responds to ER stress, is that we found that extensive exposure to BCG via intravesical treatment lowered the incidence of AD compared to alternative therapies for the same diagnosis [9]. NMIBC is the only diagnosis where patients undergo such a course of BCG, and their PBMCs may be repurposed for analysis of the UPR concerning AD. PBMC analysis has merit because peripheral immunocytes are present in the brain [37,38] and may support the protection of the aging brain [43,44], separately from their involvement in NMIBC therapy. ER stress, and consequently the UPR activation, is an early event in AD [17,45]. Therapeutic clearance of beta-amyloid aggregates with no benefit to the patients [18,19,20] indicates dysfunction in the UPR cascade or even more upstream, in the PBMCs or brain cells. The fact that numerous publications implicate the UPR in AD pathology [15,16,17,21,26,27,45,46,47,48,49] is compelling, and the fact that BCG reduces AD incidence in NMIBC makes it obvious that PBMCs should be analyzed for BCG impact on the UPR. The fate of the donors of pre- and post-BCG PBMCs to develop AD is not known, and yet the BCG impact on PBMCs is important as these immunocytes potentially support brain wellness if and when needed [43,44].

Patients suffering from NMIBC may benefit from intravesical BCG instillations and are expected to show variability in their responses corresponding to the variability of their immunogenetic gestalt, age, health factors (Table 1), and other factors that we still cannot predict. Therefore, we have compared PBMC features of ER stress response to BCG on a longitudinal basis to remove part of the individual variability from the analysis of the results. Another factor responsible for the heterogeneity of the NMIBC patients is the reported correlation of the tumor cells and their infiltrated immunocytes to express checkpoint ligands (PD-L1) of their receptor (PD-1), which raises the chance of the antitumor immunological therapy [50,51]. BCG has upregulated PD-L1 expression in PBMCs of melanoma patients, which raises the question of what kind of therapeutic effect PD-L1 expression in PBMCs has versus bladder tumors and versus brain cells in preventing AD, which must be investigated in the future. It should be stressed that the recruitment of controls of individuals not diagnosed as NMIBC receiving the same BCG treatment is unachievable.

PERK protein is one of the ER membrane resident stress sensors; PERK becomes p-PERK upon its activation, which converts its cytosol-facing domain into a protein kinase that specifically targets Ser51 of the alpha component (eIF2a) of the translation factor 2 into p-eIF2a [24,28]. The p-PERK and p-eIF2a in PBMCs of pre-BCG patients were highly abundant when expressed per total (deficient levels) PERK and eIF2a denominators, respectively (Figure 2A). In contrast, BCG treatment pushed the ratios (p-PERK/PERK and p-eIF2a/eIF2a) down and shifted them to the left by strongly increasing total PERK and eIF2a. A possible reason is that PERK and eIF2a mRNAs may translate cap-independently (which has not been shown yet in the literature). The cap-independent translation enables mRNAs to use IRES instead of the cap, which has been best studied in yeast [52,53]. The high total PERK and eIF2a is consistent with their IRES-dependent translation, ignoring the p-eIF2a-imposed limitation on cap-dependent translation, which is expected to affect the majority of cap-dependent cellular mRNA translations. Both p-PERK and p-eIF2a determine the rate of cellular protein synthesis, and their expression should not be normalized by total cellular protein to avoid the erroneous picture seen in Figure 2B versus Figure 2A. Instead, both p-PERK and p-eIF2a should be normalized by PERK and eIF2a, respectively, as normalization by total protein levels, which they determine, posits them in a “conflict of function”, so to speak. Activated p-PERK that increases p-eIF2a may cause increased expression of ATF4 that leads to increased CHOP expression [54], a dominant-negative competitor against CREB transcription factors, and thus may upregulate the expression of proapoptotic proteins [33,54,55]. BCG treatment increases Bcl2 expression, a cell survival protein [56,57], pari passu with the increased CHOP as preemptive antiapoptotic protection (Figure 3B, Table S2). Interestingly, Bcl2A1 was much lower than the canonical Bcl2 (Figure 3A), which was the opposite of the case in BCG-vaccinated melanoma patients [58]. This might be ascribed to several differences in BCG strains and administration modes. In the melanoma patients, the BCG strain obtained from the Danish Statens Serum Institute was injected intradermally, and the time interval between pre-BCG and post-BCG sampling was only 4 months. Contrarily, for the NMIBC patients, the Tice strain was used, the exposure was via the bladder, and the interval between pre- and post-BCG sampling was up to several years. The total IRE1a (Figure 4B pre-BCG) is almost zero in patient 4 because most of it is phosphorylated (activated by stress), while in patient 2 (also under ER stress), there is sufficient IRE1a and therefore also more p-IRE1a than in patient 4. After BCG treatment, IRE1a increases in both patients by a similar ratio, and so does the increase in p-IRE1a. Figure 4A (pre-BCG) shows the same dot scatter (for patients 4 and 2) as in Figure 4B, only that here the figures are related to the total protein. This leaves the IRE1a/total protein abundance ratio in a similar relative position for patients 4 and 2; patient 2 is related to less total protein than patient 4, which spans the distance between them but less so on the *y*-axis. In the post-BCG state, patient 2 has gained total protein, and patient 4 lost total protein, such that, versus IRE1a/total protein (*x*-axis, Figure 4A), they fall between the pre-BCG state, as if BCG mitigates them. They also lose height versus the p-IRE1a/total protein abundance ratio (*y*-axis, Figure 4A), which relates them to the increased total protein denominator. In Figure 4C, the post-BCG p-IRE1a values of patients 4 and 2 (red dots) are depicted on the *x*-axis to the left (reduced abundance ratio), versus the pre-BCG of patients 2 and 4, outlined by blue dots. BCG increased the activated stress sensor p-IRE1a (although it virtually decreased it relative to a higher normalizing total protein). P-IRE1a activated the XBP1 transcription factor, whose increase can be seen on the *y*-axis (Figure 4C) in response to the post-BCG p-IRE1a of patients 2 and 4 and also patients 1, 3, and 5. This is consistent with the ability of BCG to activate XBP1 splicing via p-IRE1a, which elicits part of the UPR protein recruitment [54] and helps clear misfolded proteins in PBMCs. XBP1s, in turn, increased BiP expression (Figure 5B) in patients 2 and 4. Interestingly, patient 4 had a full 3 years to express the BCG impact, followed by patient 2, with 2.5 years to express the BCG impact on the anti-apoptotic score (Figure 7B and Figure 8B).

ATF6(N) and its cooperating protein OASIS act as transcription factors shortly after their stress-sensing precursors undergo intramembranous cleavage [54], whereas XBP1 has to first undergo splicing into XBP1s at the mRNA stage by the stress sensor p-IRE1a on the XBP1u (unspliced) mRNA, before being translated. This was shown to cause a delay in XBP1s versus ATF6(N) protein expression [54] and may partially explain the low abundance of XBP1s versus ATF6(N) and OASIS, comparing Figure 4B to Figure 4A and Figure 4C. In patients 1, 2, and 3, ATF6 (the precursor of ATF6(N) is maximally cleaved/consumed for the generation of the UPR proteins important for the clearance of misfolded and aggregated ER proteins. This could occur in association with heavy smoking by patients 1, 2, and 3 (Table 1). Heavy smoking causes oxidative stress in lung tissue that causes ER stress and activation of the UPR [59]. The results show that PBMCs may also react to smoking by increasing the UPR, including ATF6 precursor expression and consumption by its conversion to ATF6(N). Contrarily, in patients 4, 5, and 6 (not recorded as heavy smokers), the ATF6 cleavage is only partial; this results in lower survival odds for the PBMCs of these patients and requires a compensatory activity for lower ATF6(N) expression. There is potential evidence of this compensatory activity in patient 4 (compare line 10 to line 9 of patient 4 in Table 2).

GAPDH upregulation by BCG, other than being part of the characteristics of innate immunity against bladder cancer, might reflect the expedited glycolytic pathway [40,60] for the benefit of brain tissue. If the hypothesis that BCG-induced PBMC aerobic glycolysis means that it may sustain astroglia, then more of the lactate end product could perhaps boost the ability of neurons to maintain their oxidative phosphorylation [61]. The missing part is the documentation of PBMCs approaching the brain from the periphery, enacting the transfer, or instructing glia to transfer lactate to neurons.

The response diversity of patients to BCG treatment is reflected in the diverse responses of the UPR proteins. One of the contributing factors to this heterogeneity seems to be the patient age during BCG therapy, their health status, and also environmental effects such as smoking. The relationship between the UPR and the patient age is observed by matching it with the score summary of UPR proteins that reflect expected cell survival (antiapoptotic) effects separated from potential cell death (proapoptotic) effects. Based on the results of Figure 7A and Figure 8A, it can be hypothesized that the benefit of BCG for resolving ER stress is maintained up to the 7th decade. This is consistent with diminishing cognition test scores with advancing age in NMIBC patients on the one hand and corrected cognition scores by BCG on the other hand [62]. The time it takes for adults to complete the full BCG treatment course (induction and maintenance) is 3 years (36 months, Figure 7B) from the treatment course, which is consistent with the peak response of GAPDH reinforced by BiP, OASIS, p-IRE1a, and Bcl2 in patient 4 (Table 2). BCG-induced activation of the glycolytic enzyme GAPDH may relate to the conversion of anaerobic to aerobic glycolysis demonstrated in type 1 diabetic patients vaccinated with BCG that showed glycemic relief only 3 years [63,64] after vaccination. The UPR protein expression changes by BCG in six patients presented here are compelling because retrospective studies show that BCG protects against AD in NMIBC patients [10,65,66]. Studies of the impact of BCG on the UPR should be expanded in more patients, including additional proteins commonly diagnostic for AD and NMIBC, which are the checkpoint receptor and ligand in PBMCs. For AD specifically, the beta-amyloid and Tau proteins and their response to BCG treatment may add to the value of BCG therapy. The limitation of this study is the lack of human brain tissue analysis, which prevented bridging the gap between the peripheral immunocytes and the changes that occur in the glia and neurons under the effect of BCG therapy.

## Figures and Tables

**Table 1:** NMIBC patients treated with intravesical BCG instillations and their pre- and post-BCG PBMC samples.

	Patient Number					
	1	2	3	4	5	6
Gender	Male	Male	Male	Male	Male	Female
Age of pre-BCG sample donation	58	57	64	47	61	82
Age of bladder exposed to BCG	58	57	64	47	61	82
Age of post-BCG PBMC sample donation	60	60	66	50	65	86
Months from the start of BCG exposure to the PBMC sample donation	24	29	28	36	50	52
Diagnostic information	Heavy smoker	Heavy smoker	Heavy smoker	Seasonal allergy	Arthritis reactive to BCG	Crohn’s disease

**Table 2:** Percent change of UPR signaling proteins in PBMCs before and after BCG bladder instillations.

Line No.	UPR Signals	Patients Number						
		1	2	3	4	5	6	
1	p-eIF2a	−61.1	−78.9	−83.3	−93.8	−8.66	−3.1	Pro-apoptotic
2	p-PERK	93.5	−89.86	−32.97	−61.9	23.59	−91.3	Pro-apoptotic
3	CHOP	58.3	62.3	64.4	93.96	81.6	12.86	Pro-apoptotic
4	BCL2	63	27.9	69.77	94.6	−96.87	−21.2	Anti-apoptotic
5	BCL2A1	58.3	-74	−58.7	59.4	18.8	−88.2	Anti-apoptotic
6	p-IRE1a	65.9	−50	84.6	99.99	58	−81.7	Anti-apoptotic
7	XBP1s	82	99.99	98.3	77.5	93	99.9	Anti-apoptotic
8	ATF6	−96.6	−98.4	−97.6	−80.6	51.3	−98.16	Anti-apoptotic
9	ATF6(N)	70.5	99.99	70.5	−13.5	−61	−65.5	Anti-apoptotic
10	OASIS	17.7	91.6	70.2	99	9.4	−50.3	Anti-apoptotic
11	BiP	20	36	−6.9	88	3.3	−49	Anti-apoptotic
12	GAPDH	5.9	93	27.3	94	56	95.8	Anti-apoptotic
13	Sum of lines 1–3	90.7	−106.46	−51.87	−61.74	96.53	−81.54	Pro-apoptotic
14	Sum of lines 4–12	286.7	226.48	257.47	518.89	131.93	−258.06	Anti-apoptotic
15	Balance between pro and anti-apoptotic effects.	196	332.94	309.34	580.13	35.4	−176.82	Balance

**Table 3:** Percent change of UPR signaling proteins/total protein abundance ratio in PBMCs before versus after BCG bladder instillations.

Line No.	UPR Signals	Patients Number						
		1	2	3	4	5	6	
1	p-eIF2a	−3	52	86.5	76.6	32	80.6	Pro-apoptotic
2	p-PERK	94.7	99.99	94.98	−54.7	99.99	43.4	Pro-apoptotic
3	CHOP	58.3	62.3	64.4	93.96	81.6	12.86	Pro-apoptotic
4	BCL2	63	27.9	69.77	94.6	−96.87	−21.2	Anti-apoptotic
5	BCL2A1	58.3	−74	−58.7	59.4	18.8	−88.2	Anti-apoptotic
6	p-IRE1a	65.9	−50	84.6	99.99	58	−81.7	Anti-apoptotic
7	XBP1s	82	99.99	98.3	77.5	93	99.9	Anti-apoptotic
8	ATF6	−96.6	−98.4	−97.6	−80.6	51.3	−98.16	Anti-apoptotic
9	ATF6(N)	70.5	99.99	70.5	−13.5	−61	−65.5	Anti-apoptotic
10	OASIS	17.7	91.6	70.2	99	9.4	−50.3	Anti-apoptotic
11	BiP	20	36	−6.9	88	3.3	−49	Anti-apoptotic
12	GAPDH	5.9	93	27.3	94	56	95.8	Anti-apoptotic
13	Sum of lines 1–3	150	214.29	245.88	115.59	181.59	136.86	Pro-apoptotic
14	Sum of lines 4–12	286.7	226.48	257.47	518.89	131.93	−258.06	Anti-apoptotic
15	Balance between pro- and anti-apoptotic effects.	136.7	12.19	11.59	403.03	−49.66	−394.92	Balance

## Data Availability

The data used in this study are available in Tables S1 and S2 of the Supplementary Section.

## Supplementary Materials

The following supporting information can be downloaded at https://www.mdpi.com/article/10.3390/cimb47060392/s1.
