# Supplementary material for: Testing Protein Stress Signals in Peripheral Immunocytes Under the Same Treatment Capable of Decreasing the Incidence of Alzheimer’s Disease in Bladder Cancer Patients
Source: Curr Issues Mol Biol. 2025 May 26;47(6):392. doi: 10.3390/cimb47060392 (PMC12191531; doi:10.3390/cimb47060392)
Supplement: Supplementary file 1 [file cimb-47-00392-s001.zip › Tables legends.pdf]

Supplementary Materials:

Table S1 legend: Transfer of the run files results from the Abby instrument. The sample indicates pre- and post-BCG of the same patients. The primary antibody discloses the antigen/protein identity. “cap”, capillary. The peak represents the measured antigen in its capillary position and MW (molecular weight). The Area of the peak represents antigen quantity expressed by an arbitrary number that is later divided by the respective total protein of each capillary, depicted separately nearby. S/N designates signal per noise of the peak.

Table S2 Legend: calculated percentage gain or loss of the [antigen/total protein] under the effect of BCG therapy for each UPR signaling protein and each patient.
